# Target-oriented motor imagery for grasping action: different characteristics of brain activation between kinesthetic and visual imagery

**DOI:** 10.1038/s41598-019-49254-2

**Published:** 2019-09-04

**Authors:** Woo Hyung Lee, Eunkyung Kim, Han Gil Seo, Byung-Mo Oh, Hyung Seok Nam, Yoon Jae Kim, Hyun Haeng Lee, Min-Gu Kang, Sungwan Kim, Moon Suk Bang

**Affiliations:** 1 Department of Biomedical Engineering, Seoul National University College of Medicine, 101 Daehak-ro, Jongno-gu, Seoul 03080 Republic of Korea; 2 Department of Rehabilitation Medicine, Seoul National University Hospital, 101 Daehak-ro, Jongno-gu, Seoul 03080 Republic of Korea; 3 Department of Rehabilitation Medicine, Konkuk University Hospital, 120-1 Hwayang-dong, Gwangjin-gu, Seoul 05030 Republic of Korea; 4 Institute of Bioengineering, Seoul National University, 1 Gwanak-ro, Gwanak-gu, Seoul 08826 Republic of Korea

**Keywords:** Cognitive control, Premotor cortex

## Abstract

Motor imagery (MI) for target-oriented movements, which is a basis for functional activities of daily living, can be more appropriate than non-target-oriented MI as tasks to promote motor recovery or brain-computer interface (BCI) applications. This study aimed to explore different characteristics of brain activation among target-oriented kinesthetic imagery (KI) and visual imagery (VI) in the first-person (VI-1) and third-person (VI-3) perspectives. Eighteen healthy volunteers were evaluated for MI ability, trained for the three types of target-oriented MIs, and scanned using 3 T functional magnetic resonance imaging (fMRI) under MI and perceptual control conditions, presented in a block design. Post-experimental questionnaires were administered after fMRI. Common brain regions activated during the three types of MI were the left premotor area and inferior parietal lobule, irrespective of the MI modalities or perspectives. Contrast analyses showed significantly increased brain activation only in the contrast of KI versus VI-1 and KI versus VI-3 for considerably extensive brain regions, including the supplementary motor area and insula. Neural activity in the orbitofrontal cortex and cerebellum during VI-1 and KI was significantly correlated with MI ability measured by mental chronometry and a self-reported questionnaire, respectively. These results can provide a basis in developing MI-based protocols for neurorehabilitation to improve motor recovery and BCI training in severely paralyzed individuals.

## Introduction

Motor imagery (MI) is a dynamic mental state during which representations of a given motor act are internally rehearsed in working memory without any overt motor output^1^. With the remarkable breakthroughs over the last two decades in the rapidly evolving field of brain-computer interface (BCI), MI has become important in rehabilitation research^2^. The neural mechanisms underlying the process of MI demonstrate functional equivalence with motor execution (ME), with the two sharing similar physiological and anatomical characteristics^3^. During MI, increased brain activity is observed at the premotor, supplementary motor, cingulate, parietal cortical areas, basal ganglia, and cerebellum, which are also the principal brain areas involved in ME^4,5^. Brain activity in motor-related areas during MI can induce neuroplasticity and reorganization of neural networks and can be adopted to control BCIs in patients with brain damage or disabilities^6,7^. In particular, for patients with severe paresis, MI may be used as the sole method to produce motor recovery of a paretic limb without overt behavioral output^8^.

MI essentially requires numerous sessions of training because many individuals cannot easily conceptualize MI^9^. It is a counterintuitive task involving a broad spectrum of processes ranging from visual imagery (VI) to kinesthetic imagery (KI), first- to third-person perspectives^10,11^, and non-target-oriented to target-oriented movements^12^. In disabled patients who do not produce overt motor output, it can be especially challenging to generate stable and continuous brain signal patterns^13^. For a wide range of MIs, an understanding of the particular brain activation patterns during specific MI can help naive MI participants modulate their brain activity effectively and proficiently for neurorehabilitation or BCI control^13,14^.

Generally, MIs can be classified into two types: VI and KI. VI involves imagining visual images of task performance in the first- (VI-1) or third-person (VI-3) perspective, and KI involves imagining the sensation produced by actually performing the task^15^. Functional magnetic resonance imaging (fMRI) studies for these three types of MI (VI-1, VI-3, and KI) have shown differential characteristics with substantial overlap among the MIs^10,16,17^. Even though the results of these studies are useful in elucidating the physiologic mechanisms for enhancing the performance of BCIs or the effect of neurorehabilitation, MI tasks only consisted of simple, non-target-oriented MIs such as finger tapping and running. Since both BCIs and neurorehabilitation aim to facilitate functionalities using instruments in daily life, the selection of these tasks may have limited application. In real-world environments, functionality in daily living can considerably depend on individual ability to use instruments that enable disabled people to perform the basic activities necessary to reside in the community^18^.

Target-oriented MI, which involves the mental rehearsal of spatial-motor actions toward a target object, has been gaining research interest because it affords the possibility of better performance in neurorehabilitation and BCIs. Previous studies using electroencephalography have shown that target-oriented MI can enhance beta-band event-related desynchronization and larger beta-band event-related synchronization with better classification performance compared to simple, non-target-oriented MI^12,19,20^. Among fMRI studies, spatial patterns of brain activity for target-oriented MI can be differentiated with ME, motor observation, or simple MI using multivoxel pattern analysis^21–24^. However, no published fMRI study has investigated the spatial differences of brain activity for subtypes of MIs including VI-1, VI-3, and KI of target-oriented movements. From a practical perspective, imagery for target-oriented movement, which can be the basis for functional activities of daily living, would be more appropriate than non-target-oriented MI as a task for motor recovery or BCI applications.

MI performance and patterns of brain activity can vary depending on an individual’s ability to form a mental representation of an action^25^. In previous studies, pre-experimental assessments for MI ability were conducted to select good imagers by using self-reported questionnaires such as the Kinesthetic and Visual Imagery Questionnaire (KVIQ) or the Movement Imagery Questionnaire^10,16^. Even though these questionnaire assessments are regarded as validated tools to estimate MI ability, they have limitations associated with the subjective assessments of participants on verbal rating scales^26^. Mental chronometry is an assessment tool to evaluate the objective temporal congruence of the movement during ME and MI and is a reliable method to assess MI^27^. Interestingly, assessment of self-reported questionnaires and mental chronometry have not shown significant relationship for any subscale or global MI ability, implying that these assessment tools reflect the multidimensional nature of MI ability and can be complementary^28^. If the particular brain areas that are correlated with the results of a self-reported questionnaire or mental chronometry during target-oriented MI can be specified in fMRI studies, this information can serve as the basis for selection of good imagers and can be used to determine the expected spatial pattern of brain activation before fMRI.

The purpose of this study was to explore the different characteristics of brain activation during target-oriented MIs between VI and KI in healthy individuals by using fMRI. We additionally aimed to investigate the correlation between MI abilities measured by a self-reported questionnaire and mental chronometry and functional brain activity using voxel-based analysis.

## Methods

### Participants

Twenty healthy, right-handed volunteers (mean age, 31.5 ± 6.5 years, 10 male) without any neurologic or psychiatric disorders were recruited in this study. Among these, two participants were excluded because one had difficulty performing a KI task and the other participant showed brain structural abnormalities on the MRI scan. Finally, eighteen participants (30.3 ± 4.3 years, nine male) were included in the analyses. The study was approved by the Institutional Review Board of Seoul National University Hospital. All participants provided written informed consent according to institutional guidelines. This study was performed in accordance with all relevant guidelines and regulations.

### Evaluation of MI ability

To evaluate the MI ability of participants quantitatively, the KVIQ-10 and sympathetic skin response (SSR), surface electromyography (EMG), and mental chronometry assessments were performed. Before fMRI scanning, participants remained in a quiet room and completed these four assessments sequentially.

The KVIQ-10 consists of five simple gestures; the participants were asked to actually perform and imagine the movements after watching a demonstration performed by the researcher. The five gestures are forward shoulder flexion, thumb to fingertips, forward trunk flexion, hip abduction, and foot tapping. The participants self-rated the clarity and intensity of the MI of these five simple gestures by using a 5-point scale from 0 (no image/no sensation) to 5 (image as clear as seeing/sensation as intense as performing the actual movement) after imagining the gestures in the visual first-person perspective and kinesthetic modalities^29^.

The SSR has been used as a reliable method to evaluate MI ability on the basis of the rationale that electrodermal activity during MI would be similar to the actual performance of the ME^30^. It enables assessment of the quality of arousal and attention during MI. For SSR measurements, surface electrodes were placed at the palmar (active) and dorsal (reference) sides of the dominant hand. After confirming no response of electrodermal activity for 30 s, the participants were instructed to conduct VI-1 or KI of hand grasping without ME. To avoid skin response adaptation, the interval between the trials was longer than 30 to 60 s, and the total duration of the test did not exceed 15 min^31,32^. The trials were repeated twice for each task, and the peak to peak amplitude (mV) and latency (ms) of the SSR were measured.

EMG was recorded to confirm that there was no actual activation of hand muscles during MI to confirm that it was distinct from ME. Additionally, a previous study reported that EMG activity during KI can be distinguished from activity induced by VI and ME on the basis of signal frequency^33^. Active and reference surface electrodes were attached at the first dorsal interosseous muscles and the first metacarpophalangeal joint, respectively.

Finally, mental chronometry was performed by asking the participants to grasp their hands for a certain amount of time (i.e., 10, 20, or 45 s). The experimenter randomly selected one of the three different time periods for hand grasping and only announced the beginning and end of the trial to the participants. After one trial of actual hand grasping, the participants were asked to perform VI-1 and KI of grasping their hands. During MIs, the participants counted the number of times they mentally grasped their hands and provided this information to the experimenter. The index of deviation from isochrony for VI-1 and KI was determined by calculating the ratio between the average duration of the actual and imagined hand grasping movements using the following formula: deviation index = abs (1 − (MI/ME))^26^.

### MI training

**Figure 1:**
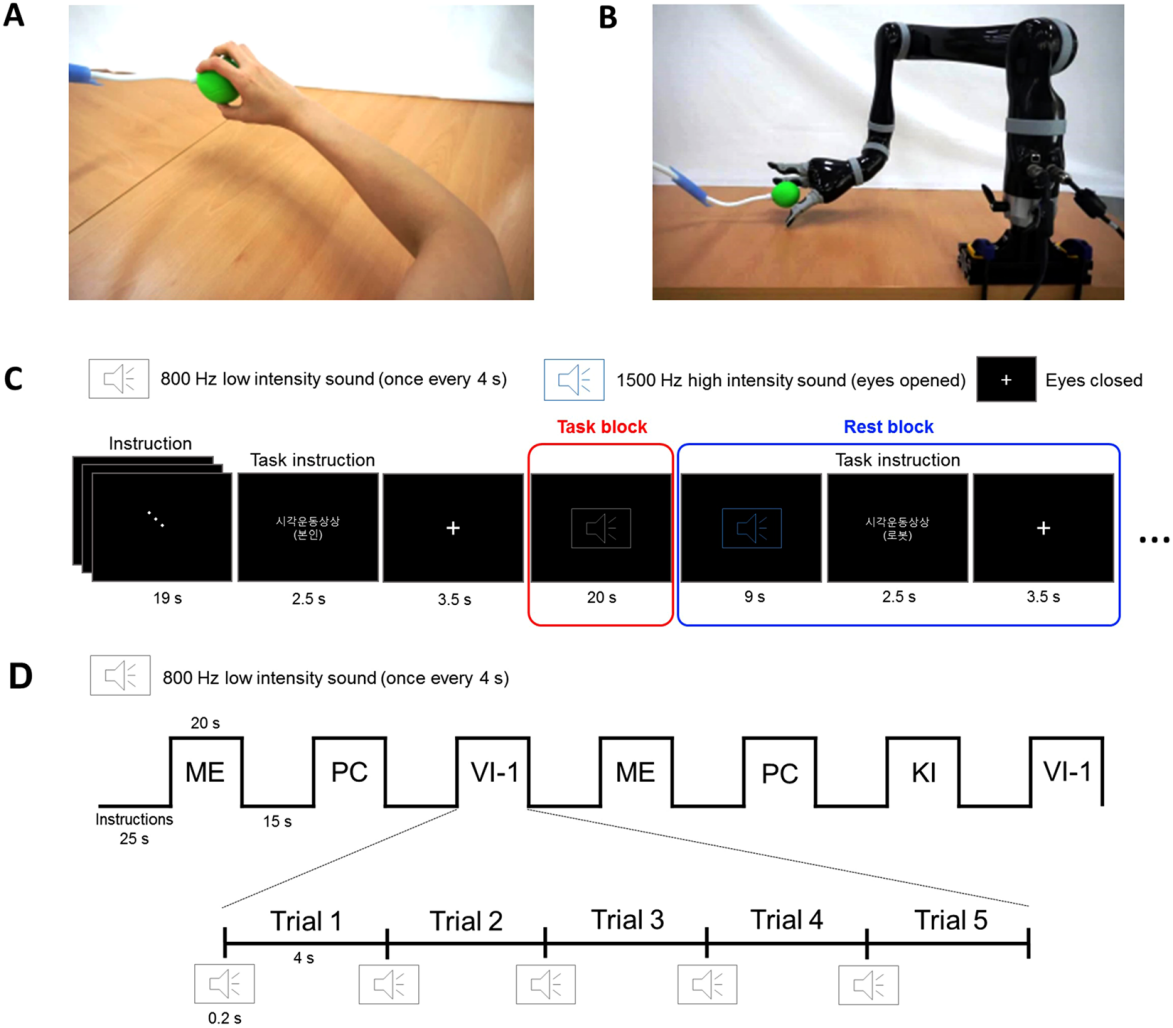
Task guidance and design for the fMRI experiment. Video guidance was provided during motor imagery training to all participants for the target-oriented visual imagery in the first-person perspective (**A**) and third-person perspective (**B**). Experimental block design and instructions during the fMRI experiment are schematically represented (**C,D**). VI-1, visual imagery in the first-person perspective; KI, kinesthetic imagery; ME, motor execution; PC, perceptual control.

The participants underwent MI training to enhance their understanding, especially for distinguishing among VI-1, VI-3, and KI. The participants were encouraged to imagine grasping and releasing the target with the right hand. To control confounding factors that may affect MI, including the shape, size, and color of a target object, view of the first-person and third-person perspectives, and surrounding experimental settings, the participants were provided video images showing the action of hand grasping as a training guide. Two types of actions were used in the video images: grasping and releasing a green, rubber ball as a target object at 4-s intervals with a right human hand in a first-person perspective view (Fig. 1A) and with a robotic hand in a third-person perspective view (Fig. 1B). Particularly, to help the participants perform target-oriented first-person perspective MIs, they were instructed to imagine the following behavioral sequences: to identify the internal representation of the target, open hand toward the target, and gently grasp and release the target.

The MI training consisted of four serial sessions. First, the participants actually grasped and released the target with their right hand every 4 s while watching the video for approximately 15 min. During the ME, they were instructed to stare at the images of a hand grasping and releasing and to sense the finger joints bending and stretching to prepare for subsequent sessions of VI-1 and KI. Second, the participants were asked to perform VI-1 while watching the guide videos for 15 min, focusing on the visual images of grasping and releasing the target using their right hand. Third, the participants were asked to perform KI for 15 min, focusing on the kinesthetic sensation of the finger joints bending and stretching toward the target. Finally, the participants were asked to perform VI-3 while watching the guide videos for 15  min, focusing on the visual images of the robotic hand grasping and releasing the target. Additionally, the participants were instructed to alternatively practice on the perceptual control (PC) condition. This resting state without any ME or MI was used as a baseline condition and the participants practiced to familiarize switching between ME/MI and PC conditions in preparation for the fMRI experiment. Whereas the MI training was conducted serially from ME, VI-1, KI, to VI-3, the participants were able to freely switch among the ME/MI/PC tasks on their own during training sessions to easily recognize the distinctions through comparison of each condition.

### Experimental procedure

Before fMRI scanning, participants were provided with experimental instructions and auditory stimuli to familiarize themselves with the experimental settings, and the researchers ensured that the participants fully understood the MI tasks to be performed during the experiment. Since no participant responses were required during the scanning, the researchers checked whether the participants were awake and whether they had made any experimental errors (e.g., missing the specific condition in a session) at the inter-session interval. The experimental paradigm was presented with the Presentation software package (Neurobehavioral Systems, Davis, CA, http://www.neurobs.com). The participants were instructed to ME and MI for grasping and releasing the target with the right hand in the same manner with training during the fMRI experiment. The target object was only used during training sessions and removed during the fMRI experiment so as not to evoke tactile sensations which can substantially influence brain activity during ME.

### Task design for the fMRI experiments

We used all five conditions as a task block: (i) ME as the physical execution of grasping and releasing the target; (ii) VI-1 of grasping and releasing the target with the participant’s hand while only focusing on VI and not on KI; (iii) VI-3 of grasping and releasing the target with the robotic hand; (iv) KI of grasping and releasing the target with the participant’s hand while only focusing on KI and not on VI; and (v) PC as the baseline condition without any ME or MI. The block design was employed in a counterbalanced manner to avoid an order effect. This study adopted a balanced Latin Square design, allocating only four conditions in one session. A total five sessions were performed for each participant and each session consisted of seven task blocks. Among the five conditions (ME, VI-1, VI-3, KI, and PC), four conditions were included in each session. Thus, three conditions were performed twice and one condition was performed once in a session. Five trials of one condition were presented per task block. A total of seven blocks (i.e., 35 trials) were performed per condition, and the inter-session interval was 3 min. One trial lasted 4 s, and a task block lasted 20 s. Rest blocks between the task blocks lasted 15 s. Notably, the baseline condition, to which ME or MI conditions were compared, was the PC condition; the rest condition was not included in the analyses. Before scanning, the participants were provided instructions via a monitor through a back-projected mirror, and scanning was started in the middle of the instructions to reduce the scanning time. During the experiment, participants were provided with auditory stimuli of high- (1500 Hz) and low-pitch (800 Hz) sounds for 0.2 s and a fixation cross for 3.5 s as instruction aids. The high-pitch sound was used to signal the participants to open their eyes and see the instruction. The low-pitch sound was repeated 5 times within each task block to announce the repetition time point of the task since 5 trials for ME or MI were conducted every 4 seconds in one task block. During the PC condition, the participants were not allowed to perform any ME or MI, but were provided with the low-pitch sounds to create an experimental environment equivalent to that of ME or MI conditions. Auditory stimuli were binaurally delivered via a headset. The fixation cross was used to signal the participants to close their eyes to remove any visual guidance or information during the tasks. Examples of the task design for the fMRI experiment are schematically presented in Fig. 1C,D.

### Post-experimental self-assessment questionnaire

After the fMRI experiment, the participants completed the self-assessment questionnaire that consisted of questions for the three types of MI. The items consisted of self-evaluated questions assessing the ease of performing MI and its consistency, which were rated on a 10-point scale from 0 (difficult/inconsistent) to 10 (easy/consistent), and for performance, vividness, controllability, interminglement among MI types, and interminglement with ME, which were rated on 5-point scales from 1 (very poor performance, no vividness, uncontrollability, no other type of imagination, and no overt movement) to 5.

### Acquisition of brain image data

Image data were obtained using a 3 T scanner (Magnetom Trio Tim; Siemens, Erlangen, Germany) with a standard 12-channel head coil. We used single-shot gradient-echo echoplanar imaging sequences, with axial acquisition of 42 slices per volume with interleaved acquisition. The image matrix was 64  × 64 mm^2^; voxel size = 1.9 mm × 1.9 mm × 3.5 mm; field of view = 240 mm; flip angle = 90°; TR = 3,000 ms, and TE = 30 ms. A total of 84 volumes were obtained per session. A high-resolution T1 image was obtained using the 3D Turbo-FLASH sequence, with sagittal acquisition of 208 slices. The image matrix was 256 × 256 mm^2^; voxel size, 1 mm^3^; field of view, 250 mm; flip angle, 9°; TR, 1,670 ms; and TE, 1.89 ms.

### Data preprocessing

Functional MR images were preprocessed using the Statistical Parametric Mapping tool (SPM12, http://www.fil.ion.ucl.ac.uk/spm/software/spm12/). Slice timing correction was applied to adjust data for interleaved slice acquisition. All functional images were realigned to the first volume to correct motion artifacts with six rigid body spatial transformations. We resliced the realigned images and created a mean functional image, which was used as a reference image for the coregistration step. To spatially normalize the functional images to the standard template of the Montreal Neurologic Institute (MNI) space (resampled at 2 × 2 × 2 mm^3^), the high-resolution structural T1 image, which was coregistered to the mean functional image, was normalized to the Tissue Probability Map template. The functional images were transformed to the MNI space by applying the deformation maps generated from the normalized procedure of structural T1 images to the template image. To increase the signal-to-noise ratio, an 8-mm full-width at half-maximum Gaussian kernel was applied to the spatially normalized functional images. The artifact detection toolbox (ART, https://gablab.mit.edu/index.php/software) was applied to detect fluctuations in BOLD signals and head motion in the functional images. Outliers were defined as volumes where the scan-to-scan differences in the global BOLD signal normalized to z-scores were greater than five and the head movement was greater than 2 mm. The total proportion of outlier volumes was 0.33% (25 out of 7,560 volumes; 84 volumes × 5 sessions ×18 participants). Eleven participants showed no outlier volumes, and the maximum number of outlier volumes (seven volumes) was observed in two participants. Therefore, we did not remove the outlier volumes to avoid any signal changes in further analyses.

### fMRI data analysis

The preprocessed fMRI data were analyzed using the general linear model, as implemented in SPM12. During the first-level individual analysis, statistical parametric maps of the *t*-statistics were calculated for each individual using a boxcar function convolved with a canonical hemodynamic response function without time and dispersion derivatives. The preprocessed individual functional images were high-pass filtered with a 128 s cutoff period to remove the effect of low-signal drift. Additionally, the six head movement parameters derived from the realignment procedure were used as regressors of no interest. FMRI data of each session were modeled with four regressors of interest, corresponding to task blocks included in a session. All brain activity during the 20 s time period of a task condition was included in the analysis. To identify the activated brain areas during MI and ME conditions, contrast images were calculated including (i) ME > PC; (ii) VI-1 > PC; (iii) VI-3 > PC; and (iv) KI > PC. Additionally, in order to investigate the activated brain areas among MI conditions, contrast images were calculated including (i) VI-1 > VI-3; (ii) VI-1 > KI; and (iii) KI > VI-3, and vice versa. The contrast images were used as the input data in group-level random-effect analyses using one-sample *t*-tests with age as a nuisance variable. Overlapping areas with brain activation were investigated using conjunction analysis (i) during all task conditions including VI-1, VI-3, KI, and ME in contrast to PC condition; (ii) during only three types of MI condition in contrast to PC; and (iii) during KI condition in contrast to VI-1 and VI-3 conditions^34^. Statistical significance was set at an uncorrected *P* < 0.001 with a cluster-based false discovery rate correction of *P* < 0.05^35^.

### Correlation between MI ability and brain activity

Multiple linear regression analysis was performed to investigate the brain regions showing associations between the results of assessment tools for MI abilities and the brain activity of MI contrasts for VI-1 > PC and KI > PC. The KVIQ scores and mental chronometry indices were used as variables of interest, and age was used as a nuisance variable. Since high KVIQ scores indicate good MI ability, a positive correlation represents increased brain activity associated with good MI ability. Conversely, the indices of mental chronometry were multiplied by −1 because low mental chronometry indices indicate good MI ability. The indices of the VI-1 and KI subcategories of 10, 20, and 45 s were averaged and used as variables of interest after multiplying them by −1. Thus, in mental chronometry, a positive correlation represents increased brain activity associated with good MI ability. Statistical significance was set at an uncorrected *P* < 0.001 with a cluster size greater than 20 voxels.

### Behavioral data analysis

The data for MI ability were analyzed using the Wilcoxon signed rank test for ordinal variables and a chi-squared test for categorical variables. The data for the post-experimental questionnaire were analyzed using the Friedman test, and post-hoc analyses were conducted using the Wilcoxon signed-rank test with a Bonferroni correction applied, resulting in a significance level set at *P* < 0.017. The average scores and deviation indices of VI-1 and KI in the KVIQ and mental chronometry, respectively, were analyzed using Pearson’s correlation coefficient. The significance level was set at *P* < 0.05. Data analysis was conducted with SPSS version 21.0 (IBM Corp., Armonk, New York, USA).

## Results

### MI ability

Table 1 shows the results of the MI ability evaluations, including the KVIQ, SSR, EMG, and mental chronometry, performed in this study. The average score of VI-1 in the KVIQ was significantly higher than that of KI (VI-1, 3.61 ± 0.78; KI, 3.03 ± 0.94, *P* = 0.014). Other evaluation methods did not show any significant differences between VI-1 and KI. The average scores of VI-1 and KI in the KVIQ were significantly correlated (*r* = 0.5088, *P* = 0.031), as were the deviation indices of mental chronometry (*r* = 0.791, *p* < 0.001). There was no significant correlation between the average scores of the KVIQ and the deviation indices of mental chronometry for either VI (*r* = 0.064, *P* = 0.801) or KI (*r* = 0.302, *P* = 0.224).

**Table 1:** Results of evaluation for motor imagery ability.

	Visual imagery	Kinesthetic imagery	*P*
Kinesthetic and Visual Imagery Questionnaire score	3.61 ± 0.78	3.03 ± 0.94	0.014*
**Sympathetic skin response**			
Response	14 (77.8)	14 (77.8)	1.000
Latency (ms)	2.04 ± 0.39	2.03 ± 0.52	0.970
Amplitude (mV)	2729.70 ± 1520.13	2637.68 ± 1344.47	0.811
**Electromyography**			
Response of motor unit action potentials	0 (0.0)	0 (0.0)	1.000
**Mental chronometry**			
Deviation index	0.46 ± 0.32	0.50 ± 0.46	0.585

Values are presented as mean ± standard deviation, or number (percent). *P < 0.05.

### Post-experimental self-reported questionnaire assessments

Table 2 shows the results of the post-experimental self-reported questionnaire assessments for the three types of MI. There were significant differences for vividness (VI-1, 3.33 ± 0.77; VI-3, 3.78 ± 0.43; and KI, 3.11 ± 0.96, *P* = 0.022) and interminglement among MI types (VI-1, 2.56 ± 0.61; VI-3, 1.61 ± 0.61; KI, 3.22 ± 0.88, *P* < 0.001). In the post-hoc analyses, vividness was significantly different between VI-1 and VI-3 (*P* = 0.011) and VI-3 and KI (*P* = 0.017). Interminglement among MI types was significantly different between VI-1 and VI-3 (*P* < 0.001) and VI-3 and KI (*P* = 0.001). Otherwise, there was no significant difference among the three types of MI.

**Table 2:** Results of self-assessment questionnaire evaluations for the three types of motor imageries.

	VI-1	VI-3	KI	*P*
Easiness^**†**^	6.61 ± 1.30	6.83 ± 1.30	6.00 ± 2.40	0.339
Consistency^**†**^	6.72 ± 1.32	7.00 ± 1.03	6.30 ± 2.02	0.361
Performance	3.56 ± 0.51	3.61 ± 0.50	3.28 ± 0.83	0.246
Vividness	3.33 ± 0.77	3.78 ± 0.43	3.11 ± 0.96	0.022*
Controllability	3.56 ± 0.70	3.83 ± 0.38	3.28 ± 0.83	0.052
Interminglement among MI types	2.56 ± 0.61	1.61 ± 0.61	3.22 ± 0.88	<0.001*
Interminglement with ME	1.67 ± 0.69	1.44 ± 0.51	2.00 ± 0.91	0.096

VI-1: visual motor imagery in the first-person perspective; VI-3: visual motor imagery in the third-person perspective; KI: kinesthetic motor imagery; MI: motor imagery; ME: motor execution.

*P < 0.05.

^†^The items for easiness and consistency of MIs were rated on a 10-point scale from 0 to 10, and other items were rated on a 5-point scale from 1 to 5.

### ME and three types of MI versus the PC condition

**Figure 2:**
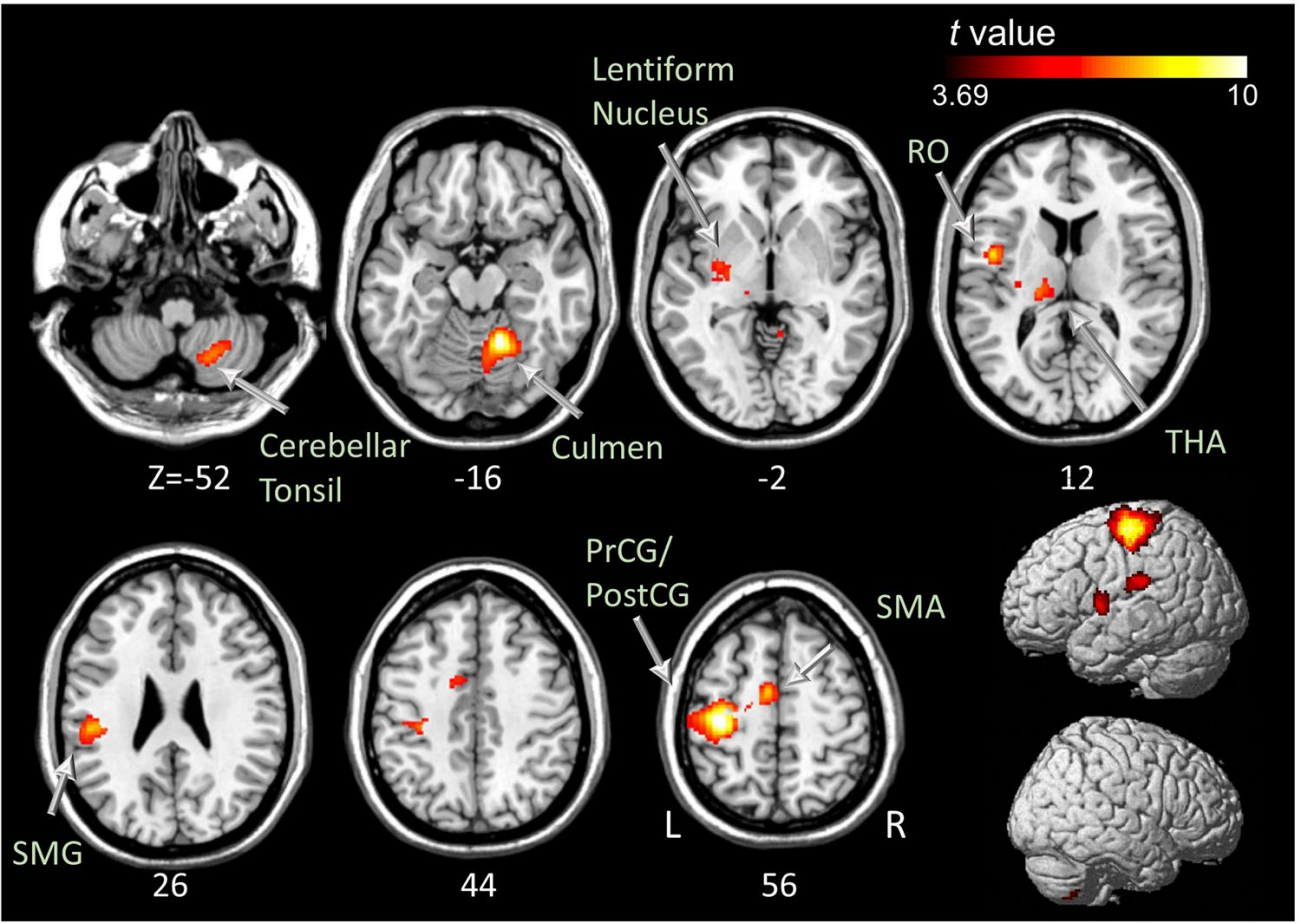
Brain regions with significantly increased activation during the motor execution versus perceptual control condition at uncorrected *P* < 0.001 with cluster-based false discovery rate correction *P* < 0.05. PostCG, postcentral gyrus; PrCG, precentral gyrus; RO, Rolandic operculum; SMA, supplementary motor area; SMG, supramarginal gyrus; THA, thalamus.

**Figure 3:**
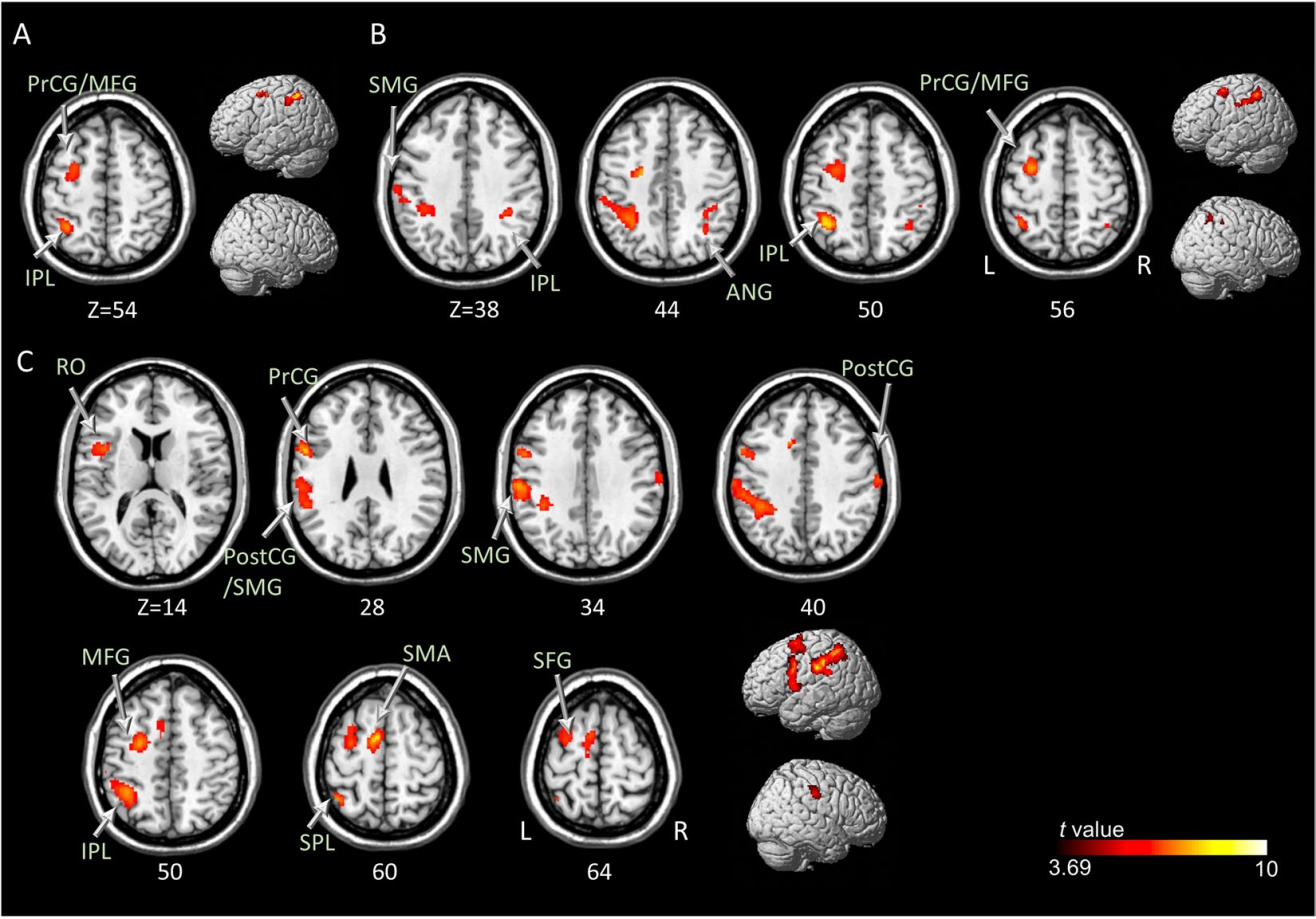
Brain regions with significantly increased activation during visual motor imagery in the first-person (**A**) and third-person (**B**) perspectives, and kinesthetic motor imagery (**C**) versus perceptual control conditions at uncorrected *P* < 0.001 with a cluster-based false discovery rate correction *P* < 0.05. ANG, angular; INS, insula; IPL, inferior parietal lobule; MFG, middle frontal gyrus; PostCG, postcentral gyrus; PrCG, precentral gyrus; RO, Rolandic operculum; SFG, superior frontal gyrus; SMA, supplementary motor area; SMG, supramarginal gyrus; SPL, superior parietal lobule.

Table 3 and Figs 2 and 3 show brain areas that were significantly activated in the ME, VI-1, VI-3, and KI conditions in comparison with the PC condition. During ME, brain activity significantly increased in the left primary motor area (BA 4), supplementary motor area (BA 6), somatosensory areas (BA 1 and BA 3), and the premotor area (BA 6; Rolandic operculum). The left supramarginal gyrus, thalamus, putamen, lateral globus pallidus, and the right cerebellum (culmen and cerebellar tonsil) also showed significantly increased brain activity (Fig. 2). During the VI-1 condition, significant brain activation was observed in the left premotor area (BA6) and the inferior parietal lobule (Fig. 3A). During the VI-3 condition, there was increased brain activity in the left premotor area (BA 6), bilateral inferior parietal lobules, left supramarginal gyrus, and right angular gyrus (Fig. 3B). During the KI condition, the brain activity of the left supplementary motor area, premotor area (BA 6; including Rolandic operculum), bilateral somatosensory areas (BA 2 and BA 3), left superior and inferior parietal lobules, and the supramarginal gyrus was increased significantly (Fig. 3C).

**Table 3:** Brain regions and coordinates of peak activations during motor execution (ME), visual imagery in the first-person perspective (VI-1), visual imagery in the third-person perspective (VI-3), and kinesthetic imagery (KI) versus the perceptual control condition (PC).

Contrast*	Region name	Hemi.	Peak T	MNI coordinates			BA
				x	y	z	
ME > PC	*Motor and premotor cortex*						
	Supplementary Motor Area	L	9.37	−10	−6	52	BA6
	Precentral Gyrus	L	8.91	−34	−18	72	BA4
	Rolandic Operculum	L	7.32	−46	0	12	BA6
	*Parietal cortex*						
	Postcentral Gyrus	L	10.89	−36	−24	56	BA3
		L	7.94	−32	−28	72	BA1
	Supramarginal Gyrus	L	6.72	−50	−28	26	BA40
	*Limbic and Subcortical regions*						
	Thalamus (Pulvinar)	L	5.63	−18	−24	10	
	Lentiform Nucleus (Putamen)	L	5.26	−32	−14	0	
	Lentiform Nucleus (Lateral Globus Pallidus)	L	5.24	−22	−8	−4	
	*Cerebellum*						
	Culmen	R	11.88	14	−54	−16	
	Cerebellar Tonsil	R	5.60	18	−64	−52	
VI-1 > PC	*Motor and premotor cortex*						
	Middle Frontal Gyrus	L	5.20	−28	2	54	BA6
	Precentral Gyrus	L	3.73	−20	−14	52	BA6
	*Parietal cortex*						
	Inferior Parietal Lobule	L	5.98	−36	−50	54	BA40
VI-3 > PC	*Motor and premotor cortex*						
	Precentral Gyrus/Middle Frontal Gyrus	L	6.26	−28	−4	56	BA6
	*Parietal cortex*						
	Inferior Parietal Lobule	L	7.38	−36	−50	50	BA40
		R	4.85	36	−42	38	BA40
	Supramarginal Gyrus	L	4.27	−60	−20	38	BA40
	Angular Gyrus	R	4.36	34	−56	44	BA39
KI > PC	*Motor and premotor cortex*						
	Supplementary Motor Area	L	7.51	−8	0	60	BA6
	Precentral Gyrus	L	6.59	−56	6	28	BA6
	Middle Frontal Gyrus	L	6.33	−26	−4	52	BA6
	Rolandic Operculum	L	5.56	−40	6	14	BA6
	*Frontal cortex*						
	Superior Frontal Gyrus	L	4.26	−28	−2	64	BA6
	*Parietal cortex*						
	Supramarginal Gyrus	L	6.46	−60	−24	34	BA40
	Inferior Parietal Lobule	L	6.27	−36	−48	50	BA40
	Superior Parietal Lobule	L	5.86	−40	−52	62	BA7
	Postcentral Gyrus	L	3.95	−46	−24	32	BA2
		R	5.27	64	−16	40	BA3

*Only the statistically significant (uncorrected P < 0.001 with cluster-based FDR threshold P < 0.05) values are reported.

**Figure 4:**
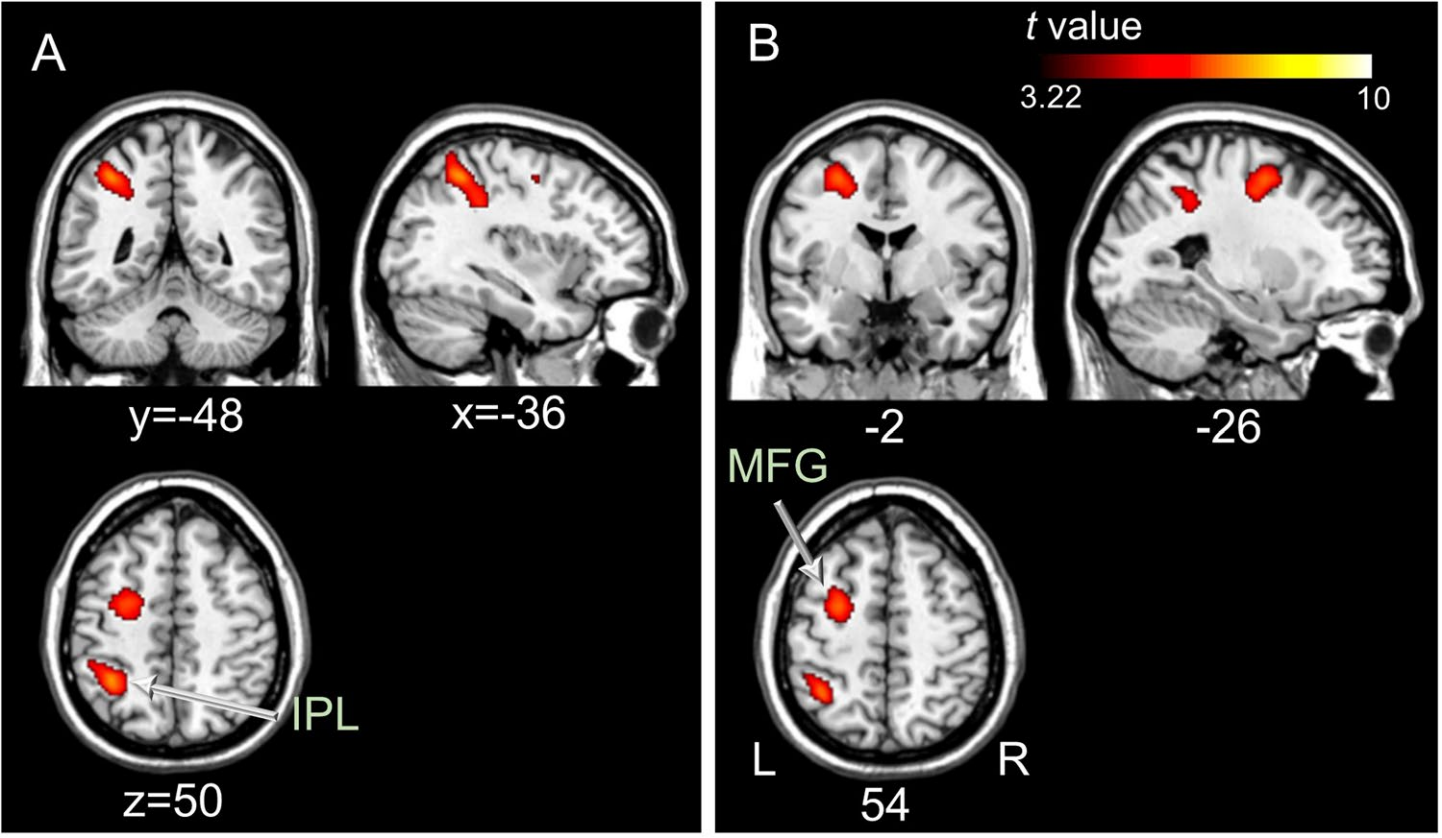
The left inferior parietal lobule (**A**) and premotor area (BA6; middle frontal gyrus, **B**) as common brain regions with significantly increased activation during the visual motor imagery in the first-person and third-person perspectives, and kinesthetic motor imagery versus perceptual control conditions at uncorrected *P* < 0.001 with cluster-based false discovery rate correction *P* < 0.05. IPL, inferior parietal lobule; MFG, middle frontal gyrus.

The common regions displaying significantly increased brain activity during all three types of MI were the left premotor area (BA 6; middle frontal gyrus) and inferior parietal lobule (Fig. 4 and Supplementary Material 1). There was no common brain area showing significantly increased brain activity during ME and the three types of MI. Additionally, the common brain area was investigated with a more lenient threshold (uncorrected *P* < 0.005, and spatial extent of voxels > 10)^16^, which showed brain areas exhibiting increased activation during ME and MI tasks (Supplementary Material 2).

### Kinesthetic vs. visual MI condition

**Figure 5:**
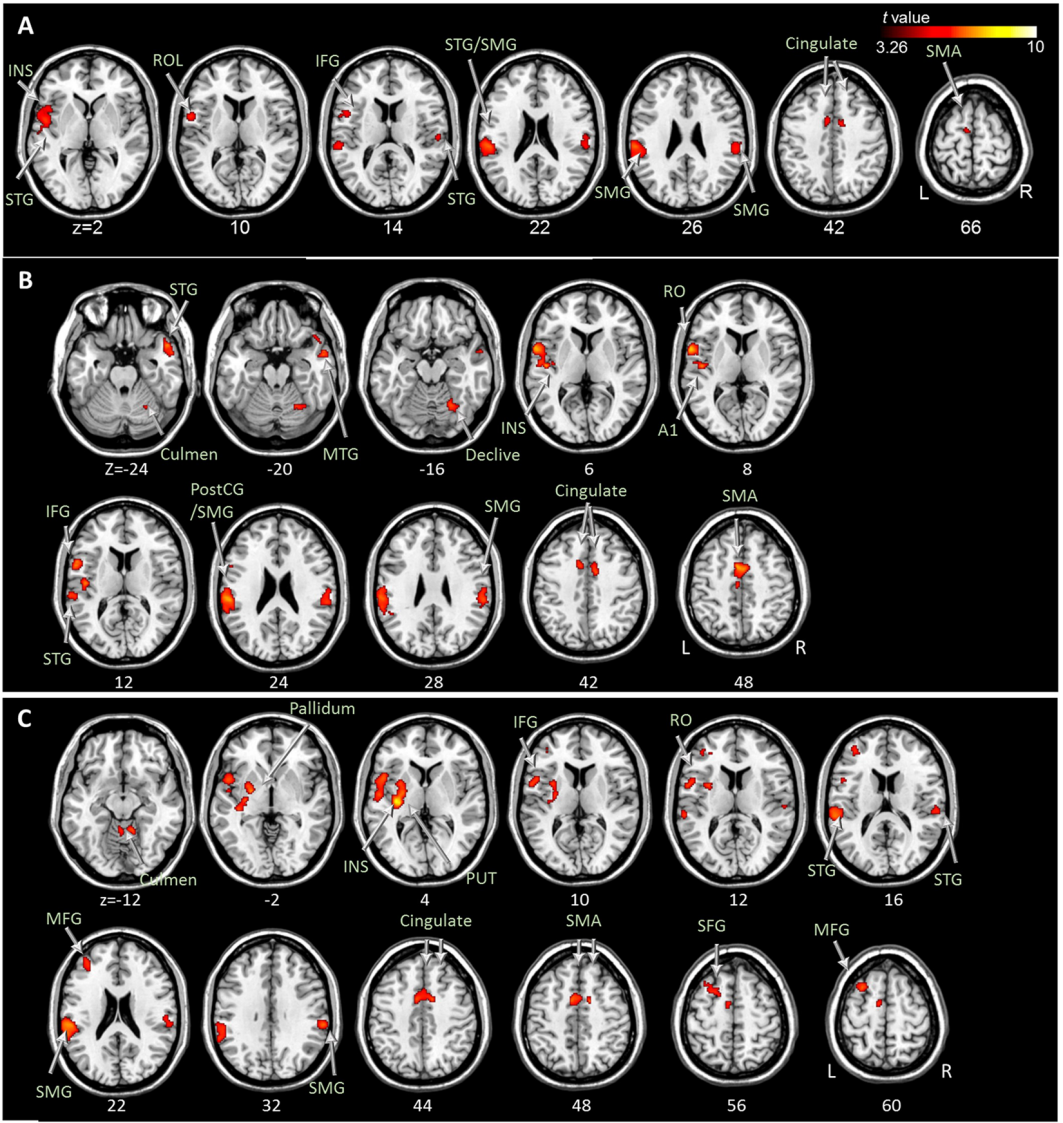
Common brain regions with significantly increased activation during both the KI > VI-1 and KI > VI-3 conditions (**A**). Brain regions with significantly increased activation during KI > VI-1 (**B**) and KI > VI-3 (**C**), respectively. All analyses were conducted at uncorrected *P* < 0.001 with cluster-based false discovery rate correction *P* < 0.05. A1, primary auditory cortex; IFG, inferior frontal gyrus; INS, insula; KI, kinesthetic motor imagery; MTG, middle temporal gyrus; PostCG, postcentral gyrus; PUT, putamen; ROL, Rolandic operculum; SFG, superior frontal gyrus; SMA, supplementary motor area; SMG, supramarginal gyrus; STG, superior temporal gyrus; VI-1, visual motor imagery in the first-person perspective; VI-3, visual motor imagery in the third-person perspective.

In the conjunction analysis among MI conditions, significantly increased brain activation was observed only in the KI > VI-1 and the KI > VI-3 contrasts; these common brain areas are shown in Table 4 and Fig. 5A, including the left supplementary motor area, premotor area (i.e., Rolandic operculum; BA 6), inferior frontal gyrus, superior temporal gyrus, insula, and bilateral supramarginal gyrus and cingulate gyrus. In comparison with VI-1, KI showed significantly increased brain activity in the left premotor area (i.e., Rolandic operculum; BA 6), somatosensory area, inferior frontal area, insula, right middle temporal area, cerebellum, including the declive and culmen, bilateral supplementary motor areas, supramarginal gyrus, superior temporal areas, and cingulate regions (Fig. 5B and Supplementary Material 3). Compared to VI-3, KI showed significantly increased brain activity in the left premotor area (i.e., Rolandic operculum; BA 6) and bilateral supplementary motor areas. Significantly increased activation was also found in the left superior, middle, and inferior frontal areas; bilateral superior temporal areas, supramarginal gyrus, left insula, left putamen and pallidum, right cingulate region, and the bilateral cerebellum (i.e., culmen) (Fig. 5C and Supplementary Material 3).

**Table 4:** Brain regions and coordinates of peak activations in the common brain areas during kinesthetic imagery versus visual imagery in the first- and third-person perspective conditions.

Region name	Hemi.	Peak T	MNI coordinates			BA
			x	y	z	
*Motor and premotor cortex*						
Supplementary Motor Area	L	3.74	−8	−6	66	BA6
Rolandic Operculum	L	4.34	−56	4	10	BA6
*Frontal cortex*						
Inferior Frontal Gyrus	L	3.78	−50	8	14	BA44
*Temporo-parietal cortex*						
Supramarginal Gyrus	L	5.49	−60	−26	26	BA40
	R	4.42	60	−28	30	BA40
Superior Temporal Gyrus	L	4.64	−52	0	2	BA22
Superior Temporal/Supramarginal Gyrus	L	5.33	−56	−28	22	BA40
	R	3.71	58	−20	14	BA40
*Limbic and Subcortical regions*						
Insula	L	3.95	−46	12	0	BA13
Cingulate Gyrus	L	4.66	−6	4	46	BA24
	R	4.27	10	4	42	BA24

*Only the statistically significant (uncorrected P < 0.001 with cluster-based FDR threshold P < 0.05) values are reported.

### Correlation between MI ability and brain activity

**Figure 6:**
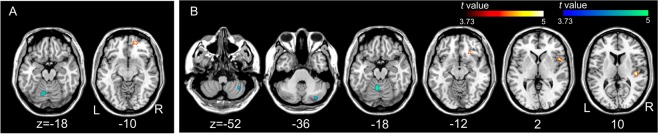
Brain regions showing significant linear positive (red to yellow) and negative (blue to green) correlations between the brain activity during kinesthetic motor imagery in the first-person perspective and the scores of the Kinesthetic and Visual Imagery Questionnaire-10 (**A**) and between brain activity during the visual motor imagery and the indices of mental chronometry (**B**). Statistical significance was set at an uncorrected *P* < 0.001 with a cluster size of more than 20 voxels.

In the KVIQ assessments, only KI showed significant linear positive and negative correlations at the right orbital superior frontal area (BA 11) and at the declive in the left cerebellum, respectively (Fig. 6A). No positive or negative correlations were observed for the VI-1 condition. In mental chronometry, only VI-1 showed significant linear positive and negative correlations in the right primary auditory cortex and inferior frontal orbitalis (BA 47), inferior frontal opercularis, and the culmen in the left cerebellum and the right cerebellar tonsil and pyramis, respectively (Fig. 6B). No positive or negative correlations were observed for KI in mental chronometry.

## Discussion

The present study aimed to investigate the activation patterns of neural activity elicited by target-oriented ME and MI. Participants performed actual movements or imagined repetitive grasp-release of a target object with the right hand for four conditions: ME, VI-1, VI-3, and KI. During the three types of MI, the common brain areas with significantly increased activity were the left premotor area (BA 6; middle frontal gyrus) and inferior parietal lobule, which did not show increased neural activity during ME. Contrast analyses among the MI conditions showed significantly increased brain activation only in the KI > VI-1 and KI > VI-3 contrasts for considerably extensive brain regions, including the supplementary motor area and insula. Neural activity in the orbitofrontal cortex (BAs 11, 47) and cerebellum during VI-1 and KI, respectively, was significantly correlated with the MI ability measured by KVIQ and mental chronometry.

### Common brain activations in the three types of MI

In this study, the left premotor area and inferior parietal lobule showed increased activation for all three types of MI. These two regions were activated irrespective of the modality (visual or kinesthetic) or perspective (first-person or third-person) of target-oriented MIs for the grasping action; those may involve processes associated with internal representation, aiming, and grasping-releasing of the target object. In a previous study, the premotor and posterior parietal areas were included as action-specific activation sites for aiming, but not for simple actions such as squeezing or flexion-extension^36^. This study indicated that motor acts, including preparation and organization of movements with specific action goals, and those involving movement simulation and object- or goal-directed tasks are the basic elements of functional organization in the premotor and posterior parietal areas, respectively. Another study also reported that the left inferior parietal lobule was critical to storing the hand postures required for interactions with target objects^37^.

However, the primary motor area was not observed as a common activated brain region in the three types of MI in this study. Although MI reportedly shares similar neural substrates of somatosensory-motor areas with ME, activation of the primary motor area during MI has been a matter of debate, as several studies have shown conflicting results^38,39^. The premotor and parietal areas are most robustly activated compared to the primary motor area during MI, implying that these areas rather than the primary motor area may be better options for implantation of an invasive BCI^40^. The results of the current study are in accordance with the previous finding in that the common brain regions activated by target-oriented MI training are the premotor and parietal areas rather than the primary motor area, regardless of MI type. Identification of activation sites and a thorough understanding of their physiological roles during MI training may allow proper site selection of electrode positioning for BCI implantation and could enhance the controllability of the BCI.

### Kinesthetic MI

Kinesthesia may include all levels of the organization of movements, including muscle tone, synergies, goal-directed movements, and action^41^. It can necessitate mental representation for generating, integrating and perceiving kinesthetic sensory signals from position and planned action of the limb without actual movement and sensory input^42,43^. In the context of neurorehabilitation, KI, rather than VI, has been recommended to promote better motor recovery and improve functionality in patients with neurologic diseases^44,45^. In the current study, overlapping brain regions with increased neural activation in the KI > VI-1 and KI > VI-3 conditions included the supplementary motor area, Rolandic operculum, inferior frontal gyrus, supramarginal gyrus, superior temporal gyrus, insula, and cingulate gyrus. These brain regions, which are possibly engaged in the neural process of KI, were activated more extensively than has been observed previously^10,16^; it can be attributed to the complexity of the MI task, in that KI with target-oriented movement might demand more extensive neural recruitment compared to simple KI. Particularly, these brain regions included the supplementary motor area and insula, the activation of which occurs after MI training in stroke pateints^46^. The supplementary motor area is a secondary motor area that is heavily interconnected with the primary motor area; it was believed to be involved in planning and pacing complex motor activities^47^. Even though the mechanism is unclear, it may exert a suppressive influence over the primary motor area during KI by actively inhibiting the ME of the represented motor actions^48^. From the perspective of BCIs, the finding that KI induced increased activation of the supplementary motor area compared to VI can be applied to MI-based BCIs, as the activation patterns of the supplementary motor area showed great predictive ability for imagined movement^49^. In addition, activation of the insula may be involved in the integration of multimodal sensory signals for the generation of simulated actions, attributable to the kinesthetic components of MI^46^. The insula can serve as the interface between body awareness and movement during KI by processing and integrating a wide range of sensory signals arising from the body to internally generate intentional movement^50^. Further study is necessary to reveal the exact neural process underlying brain activation elicited by KI and to optimize KI-based protocols for BCI training and neurorehabilitation in order to facilitate the efficiency of motor recovery.

### Visual MI

Intriguingly, no brain region in the occipital lobe was activated during VI-1 and VI-3 in contrast with the PC condition. This result may not be attributable to the difficulty of VI, since the subjective assessments including the KVIQ and post-experimental questionnaire showed that MI ability, vividness, and interminglement among MI types during VI are significantly better than those during KI. In fact, the involvement of the primary visual cortex and adjacent areas in VI has been a topic of debate in the literature^51,52^. Even though previous studies have reported the predominant activation of occipital regions during VI^10,16^, other published studies have indicated that the parietal regions (BA 7 and 40) can be highly activated with slight or no activation of the occipital regions, including the primary and secondary visual cortices^17,51,52^. In this study, VI-1 and VI-3 showed increased activity in the inferior parietal lobule. The inferior parietal lobule has been noted to play a crucial role in the processing of visual information: maintaining attention on the targets and responding to salient information and new events, which can be relevant to the tasks of VI-1 and VI-3^53^. Particularly, in the present study, brain activity was increased in the left inferior parietal lobule during VI-1 and VI-3, but only in the right inferior parietal lobule during VI-3. These results were consistent with a previous study which demonstrated that the right inferior parietal lobule can have a determinant role in high-order body or self-representation, and abnormal responses in this region can cause the misattribution of self-generated actions to external entities^54,55^. In contrast, the first-person perspective may result in strong left-hemispheric activation of the inferior parietal lobule in the current study, which may indicate that this region has a prominent role in programming self-movements^55^.

### Visual and kinesthetic MI abilities

A whole-brain regression analysis for MI ability and the activity levels of brain regions showed considerably different results for the KVIQ and mental chronometry. Analysis of brain activity in the VI-1 > PC condition showed only significant correlations with the indices of mental chronometry, while brain activity in the KI > PC condition showed only significant correlations with the KVIQ scores. These findings are consistent with results showing no significant correlation between the KVIQ scores and mental chronometry indices for both VI-1 and KI in this study. A previous study reported similar findings in that the self-reported questionnaire results for MI abilities and mental chronometry data did not show significant relationship for any subscale or global MI ability^28^. This may indicate that the KVIQ and mental chronometry reflect different dimensions of MI ability for KI and VI-1, respectively, and can serve complementary roles in differentiating between visual and kinesthetic MI in fMRI studies.

Interestingly, despite the different types of target-oriented MIs, good imagers for KI in the KVIQ and for VI-1 in mental chronometry showed increased brain activity at the orbitofrontal cortex (BAs 11, 47) and decreased brain activity at the cerebellum. This different aspect of brain activation in the two brain areas was also reported by a previous study, which reported that healthy participants showed increased activity in the orbitofrontal cortex and decreased cerebellar activity after intensive MI training^56^. The orbitofrontal cortex can be involved in learning or recalling sequential motor tasks with synchronization to external stimuli at regular intervals^55–57^ During MI tasks in the fMRI experiment, the participants imagined hand grasping and releasing, sequentially, at regular intervals, accompanied by auditory stimuli, which may have led to activation of the orbitofrontal cortex in the participants. Conversely, we observed that the greater the MI abilities were, the more the cerebellar activity decreased. Even if the cerebellum plays a pivotal role in acquiring new motor routines, particularly in the early stages of the acquisition process^58^, cerebellar activity can decrease as participants reach some level of automatization on the motor sequence learning task^56^. This inhibitory effect of the cerebellum on MI was suggested by a previous study as was its opposing effect on the contralateral motor cortex during ME^59^. The physiological mechanism underlying brain activity at the orbitofrontal cortex and cerebellum during MI should be investigated in further studies, which will be helpful in distinguishing good imagers for specific types of MI with distinct patterns of brain activity using simple evaluation methods such as the KVIQ and mental chronometry, without neuroimaging modalities.

This study had several limitations. First, no participant was excluded based on the results of MI ability assessments; thus, poor imagers might have been included in this study. However, even though KVIQ, SSR, EMG, and mental chronometry were conducted to evaluate MI ability, classification of good and poor imagers can be an arbitrary distinction based solely on the results of a self-reported questionnaire assessment as in previous studies^10,16^. Since individuals who need to be trained in using MI modalities can show low levels of MI abilities due to cognitive impairment as a result of brain injuries, inclusion of all participants irrespective of MI abilities may yield more generalized results that can be applied in actual clinical practice. Second, EMG was not measured during fMRI data acquisition to confirm no actual movement during MI sessions. Instead, EMG measurements were obtained during MI training sessions before the fMRI experiment to confirm that no motor unit action potential was observed during MI as the participants were being educated. After the EMG measurement, the participants were trained to simulate the three types of MI without any actual movement of their hand for 1 h to adapt themselves in the fMRI sessions. Third, it was practically difficult to completely exclude visual component from KI during the fMRI experiment. The post-experimental self-assessment questionnaire showed a significantly high rate of interminglement among other MI types during KI compared to VI-1 and VI-3. Further study will be necessary to quantitatively measure and control the visual component during KI by providing feedback on the participants during MI training. Fourth, both KVIQ-10 and mental chronometry were performed only for VI-1 and KI in order to perform a consistent assessment. KVIQ-10, which should be conducted strictly according to the administration procedures, is an evaluation method solely for VI-1 and KI, and it was difficult to adapt for VI-3.

In conclusion, the present study revealed the different characteristics of brain activity during target-oriented MIs in healthy individuals by using fMRI. The common brain regions activated during the three types of MI were the premotor area and inferior parietal lobule, irrespective of the different modalities and perspectives of target-oriented MIs. Based on contrast analysis between each MI condition, significantly increased brain activation was observed only in the contrast of KI versus VI-1 and KI versus VI-3 for considerably extensive brain regions, including the supplementary motor area and insula. Neural activity in the orbitofrontal cortex and cerebellum during VI-1 and KI was significantly correlated with MI abilities measured by mental chronometry and the KVIQ. The characteristics of brain activity during target-oriented MIs can provide a basis to develop MI-based protocols for neurorehabilitation to facilitate the efficiency of motor recovery and BCI training for severely paralyzed individuals. Further study is necessary to reveal the long-term training effects for more complex tasks of each type of MI to advance training protocols in the field of BCI and neurorehabilitation.

## Supplementary information

Supplementary Table 1-3
