# Supplementary material for: Target-oriented motor imagery for grasping action: different characteristics of brain activation between kinesthetic and visual imagery
Source: Sci Rep. 2019 Sep 4;9:12770. doi: 10.1038/s41598-019-49254-2 (PMC6726765; doi:10.1038/s41598-019-49254-2)
Supplement: Supplementary file 1 — Supplementary Table 1-3 [file 41598_2019_49254_MOESM1_ESM.pdf]

Title: Target-oriented motor imagery for grasping action: different characteristics of brain activation between kinesthetic and visual imagery

Authors names and affiliations:

Woo Hyung Lee, MD[1,†]

Eunkyung Kim, PhD[2,†]

Han Gil Seo, MD, PhD[2]

Byung-Mo Oh, MD, PhD[2]

Hyung Seok Nam, MD, PhD[2]

Yoon Jae Kim, PhD[1]

Hyun Haeng Lee, MD[3]

Min-Gu Kang, MD[2]

Sungwan Kim, PhD[1,4,*]

Moon Suk Bang, MD, PhD[2,*]

[1]Department of Biomedical Engineering, Seoul National University College of Medicine, 101 Daehak-ro, Jongno-gu, Seoul, 03080, Republic of Korea

[2]Department of Rehabilitation Medicine, Seoul National University Hospital, 101 Daehak-ro, Jongno-gu, Seoul, 03080, Republic of Korea

[3]Department of Rehabilitation Medicine, Konkuk University Hospital, 120-1 Hwayang-dong, Gwangjin-gu, Seoul, 05030, Republic of Korea

[4]Institute of Medical and Biological Engineering, Medical Research Center, Seoul National University, 101 Daehak-ro, Jongno-gu, Seoul, 03080**,** Republic of Korea

Corresponding authors:

(1) Moon Suk Bang, MD, PhD

Department of Rehabilitation Medicine, Seoul National University Hospital, Seoul National University College of Medicine, 101 Daehak-ro, Jongno-gu, Seoul, 03080, Republic of Korea

Phone: +82-2-2072-2619

Fax: +82-2-743-7473

E-mail: msbang@snu.ac.kr

(2) Sungwan Kim, PhD

Department of Biomedical Engineering, Seoul National University College of Medicine, 101 Daehak-ro, Jongno-gu, Seoul, 03080, Republic of Korea

Institute of Medical and Biological Engineering, Medical Research Center, Seoul National University, 101 Daehak-ro, Jongno-gu, Seoul, 03080, Republic of Korea

Phone: +82-2-2072-3126

Fax: +82-2-745-7870

E-mail: sungwan@snu.ac.kr

†Co-first authors: these authors contributed equally to this work

*Co-corresponding authors: these authors contributed equally to this work

Supplementary Table 1. Brain regions and coordinates of peak activations in the common brain areas during visual imagery in the first- and third-person perspectives and kinesthetic imagery conditions versus the perceptual control condition

Region name	Hemi.	Peak T	MNI coordinates			BA
			x	y	z	
Inferior Parietal Lobule	L	6.05	-36	-48	50	BA40
Middle Frontal Gyrus	L	5.25	-26	-2	54	BA6

Supplementary Table 2. Brain regions and coordinates of peak activations in common brain areas during the motor execution and three types of motor imagery conditions versus the perceptual control condition

Region name	Hemi.	Peak T	MNI coordinates			BA
			x	y	z	
Motor and premotor cortex						
Supplementary Motor Area	L	3.30	-12	-4	60	BA6
Precentral Gyrus	L	3.25	-30	-6	60	BA6
Parietal cortex						
Postcentral Gyrus	R	3.43	66	-14	38	BA3
Inferior Parietal Lobule	L	2.76	-40	-44	52	BA40

Supplementary Table 3. Brain regions and coordinates of peak activations in brain areas during the kinesthetic imagery condition versus visual imagery in the first- and third-person perspective

Contrast	Region name	Hemi.	Peak T	MNI coordinates			BA
				x	y	z	
KI > VI-1	*Motor and premotor cortex*						
	Rolandic Operculum	L	6.77	-60	6	8	BA6
	Supplementary Motor Area	L	6.31	-4	4	48	BA6
		R	6.01	2	2	48	BA6
	Frontal cortex						
	Inferior Frontal Gyrus	L	5.66	-56	10	12	BA44
	Temporal cortex						
	Superior Temporal Gyrus	L	6.12	-48	-14	6	BA22
		L	4.76	-64	-28	12	BA42
		R	5.60	50	14	-24	BA38
	Middle Temporal Gyrus	R	5.40	56	2	-20	BA21
	Parietal cortex						
	Supramarginal Gyrus	L	6.35	-58	-26	24	BA40
		R	4.87	60	-30	28	BA40
	Postcentral Gyrus	L	4.67	-60	-16	24	BA3
	Limbic and Subcortical regions						
	Cingulate Gyrus	L	4.67	-6	-16	46	BA24
		R	5.17	10	0	42	BA24
	Insula	L	4.45	-40	-12	6	BA13

	Cerebellum						
	Declive	R	4.82	24	-62	-16	
	Culmen	R	4.72	24	-54	-12	
KI > VI-3	*Motor and premotor cortex*						
	Supplementary Motor Area	L	5.34	-8	2	48	BA6
		R	6.21	14	2	48	BA6
	Middle Frontal Gyrus	L	5.34	-28	16	60	BA8
	Rolandic Operculum	L	4.81	-54	6	12	BA6
	Frontal cortex						
	Inferior Frontal Gyrus	L	4.65	-50	10	10	BA44
	Middle Frontal Gyrus	L	4.61	-36	44	18	BA10
	Superior Frontal Gyrus	L	4.41	-22	12	56	BA6
	Temporal cortex						
	Superior Temporal Gyrus	L	5.62	-60	-32	16	BA42
		L	4.00	-62	-46	18	BA22
		R	4.36	58	-24	14	BA42
	Pareital cortex						
	Supramarginal Gyrus	L	6.00	-58	-28	22	BA40
		R	5.40	62	-30	32	BA40
	Limbic and Subcortical regions						
	Insula	L	7.88	-34	-16	4	BA13
	Lentiform Nucleus (Putamen)	L	7.30	-30	-18	4	
	Lentiform Nucleus (Pallidum)	L	5.87	-24	-4	-2	
	Cingulate Gyrus	R	5.18	12	4	44	BA24

Cerebellum					
Culmen	L	4.87	-2	-48	-8
	R	4.92	12	-48	-12
